# Exploring the effect of diet programs on the behavior of Sudanese children with autism and the prevalence and association of gastrointestinal symptoms: a multi-center cross-sectional study

**DOI:** 10.1097/MS9.0000000000002526

**Published:** 2024-09-04

**Authors:** Sara Elawad, Shaima Omer Mohamed Elawad, Mohamed H. Elbadawi, Wafa Yousif Abdalla Sosal, Leena Mohamed Khalid, Doaa Rabeie Hassan AbdEldaim, Lina Hemmeda, Khabab Abbasher Hussien Mohamed Ahmed, Ghassan E. Mustafa Ahmed

**Affiliations:** Faculty of Medicine, University of Khartoum, Khartoum, Sudan

**Keywords:** autism, autistic children, behavioral assessment, food allergy, food selectivity, gastrointestinal symptoms, prevalence

## Abstract

**Background and aims:**

Autism spectrum disorder (ASD) is a neurodevelopmental condition characterized by social difficulties, speech and nonverbal communication issues, and restricted behaviors. Nutritional issues, such as food allergies and intolerances, can affect children with ASD. This study aimed to evaluate the impact of diet programs on ASD behavior and gastrointestinal symptoms, which would be considered as a starting point to increase the family’s knowledge about how to practice healthy and suitable dieting for their children.

**Materials and methods:**

The study was a cross-sectional observational study on 45 children with an autism spectrum disorder in four centers aged 2–18 in Khartoum state. Data was collected through an interview questionnaire, which included sociodemographic, diet, gastrointestinal, and behavioral assessments. The data was analyzed using SPSS to find the correlation between the various variables. Independent *t*-test, Analysis of Variance, and Mann–Whitney test were used in univariant analysis to assess the association between study variables, while multiple linear regression was used in multivariant analysis for the same purpose

**Results:**

The study involved a large number of children with autism spectrum disorder (ASD), with 80% being male and 73.3% being school-aged. Most had parents as guardians and 71.1% were in intermediate financial status. About 20% were overweight or obese, and 57.8% did not have a specific diet program. Food selectivity was prevalent, with 22.7% having allergies to milk and wheat. ASD children experienced vomiting, gastric reflux, abdominal pain, and changes in stool characteristics. A significant link was found between financial status, behavioral status and gastrointestinal changes. High financial status was significantly different from intermediate and low statuses based on behavioral changes. However, no significant association was found in multivariant analysis.

**Conclusion:**

The study found that ASD children generally have good nutritional health, with a higher degree of dietary selection. The financial status of guardians significantly influenced behavioral and gastrointestinal changes in the children. Further interventional studies are recommended to assess the direct impact of diet programs on these symptoms.

## Introduction

Highlights

- Social difficulties, problems with speech and nonverbal communication, and limited activities are the hallmarks of autism spectrum disorder (ASD), a neurodevelopmental illness.
- Children diagnosed with ASD may have nutritional problems, including food allergies and intolerances. The goal of this study was to assess how food plans affected the behavior of people with ASD and their gastrointestinal issues.
- The 45 children with autism spectrum disorder in four centers, ages 2–18, were the subjects of this cross-sectional observational research conducted in the state of Khartoum.

Autism spectrum disorder (ASD) is a complex neurodevelopmental condition that involves persistent problems and difficulty in social interactions, speech, and nonverbal communication, and restricted/repetitive behaviors compared to its matched child^1^. In 2018, the CDC estimated that ~1 in 59 children are diagnosed with an autism spectrum disorder (1 in 37 boys, 1 in 151 girls) worldwide^2^. It has been observed that autism is indeed a common disorder and its incidence is rising in all parts of the world and -to the study’s concern – here in Africa. One study, for example, that involved two North African countries documented a high frequency of ASD at 11.5 and 33.6% among African children with developmental disorders. Other studies conducted among children of African descent have reported a high occurrence of ASD^3^.

Manifestations of the disorder vary greatly depending on the severity of the autistic condition, developmental level, and chronological age; hence, the term spectrum. The essential diagnostic features/criteria of autism spectrum disorder are persistent impairment in reciprocal social communication and social interaction (Criterion A), and restricted, repetitive patterns of behavior, interests, or activities (Criterion B). These symptoms are present from early childhood and limit or impair everyday functioning (Criteria C and D). Diagnoses are most valid and reliable when based on multiple sources of information, including clinician’s observations, caregiver history, and when possible, self-report. Autism can be reliably diagnosed as early as age 2^2^.

Children with autism face several nutritional challenges. They are at risk for food allergies, food intolerances, selectivity, feeding problems, and nutrient deficiencies^4^. They may suffer from GI symptoms, including abdominal pain, chronic diarrhea, constipation, vomiting, gastroesophageal reflux, intestinal infections, and increased intestinal permeability^5^.

A special diet has a great and serious effect on the lives of autistic children. A Gluten-free, casein-free (GFCF) diet was found to significantly improve social isolation, eye contact, mutism, learning skills, hyperactivity, stereotypic activity, and panic attacks in a large number of patients^6^. Furthermore, many studies revealed that many children with ASD often have deficiencies in lactase and other digestive enzymes, and food sensitivities-especially to gluten and casein-and therefore may benefit from digestive enzymes, healthier diets, and/or gluten-free, casein-free diets^7–10^.

With the rise in ASD prevalence and the scarcity of information on ASD and their nutrition in Sudan, this study aimed to evaluate the effects of diet programs on the behavior of children with autism and the prevalence and association of gastrointestinal symptoms in Khartoum state, Sudan, 2022. This research is considered a starting point to increase the family’s knowledge about how to practice healthy and suitable dieting for their children. With a special focus on Sudanese food and diet and how it affects children with ASD, to our knowledge, no research published in Sudan has studied this issue.

## Methods

This is an observational cross-sectional study with both descriptive and analytical components, which was conducted in Khartoum state, the capital of Sudan. Khartoum state is the smallest state by area (22 142 km^2^). However, it is the most populous (5 274 321 according to the 2008 census). Khartoum is divided into three large cities: Khartoum proper, Omdurman, and Bahri^11^. Special education centers where autistic children learn and get followed up are located in these cities. The study included at least one center from each city. Cluster random sampling was used to select the centers, and the data was collected in the period from the 1st of January to the 1st of February 2022. All Children with ASD between 2 and 18 years old, residing in Khartoum state and visiting selected centers to follow up, and who were otherwise healthy were recruited until the sample size was fulfilled. The sample size was calculated using Cochran’s formula^12^


$$n=\frac{z^{2}\mathrm{pq}}{e^{2}},$$

where *n*: sample size.

*z*: standard error associated with the chosen level of confidence (95%).

*p*: proportion of the population (prevalence).

*q* =1-p.

*e*=accepted sample error.

The prevalence of autism in Sudan and Africa was not well established, but according to a study conducted in Nigeria, it is found to be 2.3%^13^. With an accepted sample error of 5%, and a 20% nonresponse rate the final sample size (*n*) was set at 45.

Data was collected through an interview questionnaire with patients’ families. The questionnaire was structured with closed and open-ended questions formed in the light and guidance of the Autism Spectrum Disorder Evaluation Scale (ASDES) by Tamara J. Arthaud, PhD^14^. Considering this target group and their context, questions were chosen, omitted, and modified for the final questionnaire, which was piloted and assessed for clarification and validation by a social worker who was provided by the Department of Community Medicine University of Khartoum, and who had experience with developing questionnaires. The questionnaire consisted of four sections: the first section includes the sociodemographic characteristics of the children and their guardians. The rest of the sections contained questions for diet assessment, gastrointestinal symptoms assessment, and behavioral assessment.

Questionnaires were refined and managed carefully, and completeness was checked before data entry. Data generated on the questionnaires were numbered and entered for analysis using Statistical Package for Social Science (SPSS) version No. 26 software for statistical analysis. Frequency tables, percentages, and figures were used to describe the data. Independent *t*-test, Analysis of Variance, and Mann–Whitney test were used in univariant analysis to assess the association between study variables, while multiple linear regression was used in multivariant analysis for the same purpose.

The work has been reported in accordance with strengthening the reporting of cohort, cross-sectional, and case–control studies in surgery (STROCSS) guidelines^15^.

## Results

### Sociodemographic characteristics of the participants

Forty-five participants were targeted in this study. Four-fifths of them were males (80%). The majority of the children were within the school age (6–15 years old) (73.3%), having mainly 1st and 2nd order of birth in the family (40 and 28.9%, respectively). More than half of the sample had both of their parents as their current guardians (60.0%) living in intermediate level of financial status (71.1%). 66.7% of the children had a moderate level of activity, while almost four fifth did not have any other medical condition (84.4%). The majority of the respondents (82.2%) reported not having a family history of autism (further details in Table 1).

**Table 1:** The table shows the sociodemographic characteristics of the participants and their *P*-values for behavioral change scales and GIT symptoms frequency scale (*N*=45)

The characteristics	Frequency	(%)	Behavioral change scale (Mean±SD)	*P* a	GIT symptoms frequency scale (Mean±SD)	*P* b
Sex:				0.400		0.126
Male	36	80%	30.24±6.88		1.42±2.55	
Females	9	20%	33.25±1.50		3.63±4.06	
Age groups				0.635		0.315
Preschool age (3–5 years old)	12	26.67%	29.71±4.07		0.67±1.07	
School-age (6–15 years old )	33	73.33%	31.11±7.18		2.25±3.32	
Residency:				0.111		0.681
Omdurman locality	16	35.6%	31.14±6.87		1.56±2.80	
Bahri locality	19	42.2%	31.50±6.57		1.11±1.81	
Khartoum locality	10	22.2%	28.67±6.22		3.50±4.28	
Number of Siblings	2.67^c^	1.75^d^		e		e
Order of the child:				e		e
1st born	18	**40.0%**	32.55±7.22		1.41±2.24	
2nd born	13	28.9%	29.86±6.09		2.46±3.91	
3rd born	7	15.6%	33.33±4.04		0.86±2.27	
4th born	5	**11.1%**	25.25±2.99		2.40±3.36	
5th born	1	2.2%	0.00±0.00		0.00±0.00	
6th born	1	2.2%	0.00±0.00		6.00±0.00	
The financial status of the guardian :				0.018*		0.833
High	**3**	6.7%	41.50±4.95		2.00±3.46	
Intermediate	32	71.1%	30.65±4.96		1.94±3.05	
Low	10	22.2%	27.33±7.28		1.40±2.80	
Who is the guardian of the autistic child?				0.077		0.611
Father	7	15.6%	28.20±8.79		4.00±4.73	
Mother	9	20.0%	30.50±3.11		1.00±2.14	
Both	27	60.0%	30.14±5.22		1.41±2.37	
Other	2	4.4%	41.50±4.95		3.00±4.24	
The level of activity in autistic children:				e		e
High	14	31.1%	28.60±5.37		1.77±2.62	
Moderate	30	66.7%	31.11±6.81		1.90±3.17	
Low	1	2.2%	34.00±0.00		0.00±0.00	
Is there any family member affected by autism?				0.964		0.501
No	37	82.2%	30.75±6.49		1.67±2.94	
Yes	8	17.8%	30.60±6.80		2.50±3.16	
Does the child have any other medical condition?				0.263		0.706
No	38	84.4%	31.45±6.14		1.65±2.75	
Yes	7	15.6%	27.80±7.33		2.71±4.03	

a The scale for behavioral change in autistic children (using ANOVA and independent *T*-test).

b The scale for the frequency of GIT symptoms of Autistic children (Using ANOVA and Mann–Whitney test).

c Mean of the variable.

d SD.

e Univariate analysis was not applicable.

* Significant result (*P*-value >0.05).

### Nutritional characteristics of the participants

**Figure 1:**
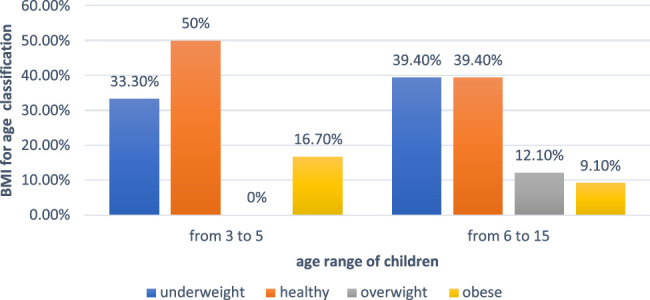
The bar chart shows the nutritional status using BMI for age and its distribution between two age groups preschool (from 3 to 5) and school-age group (from 5 to 15) for autistic children among participants in 2022 in Khartoum state (*N*=45).

Figure 1 shows the distribution of the BMI across the two age groups and it illustrates that the highest BMI scaled is within the 3–5 age group (almost BMI of 50%). Only a fifth of the participants were overweight or obese (20.0%). Almost more than half of autistic children (57.8%) do not have a specific diet program, even though more than half of the sample (54.5%) believe in the effect of diet programs on behavior. More than half of the children reported having food selectivity (57.56%) with almost less than a third of the sample having allergies to milk and wheat (22.2% for both). Moreover, more than half of the autistic children are not given any autism supplements (57.8%). Only a small portion of the participants (4.5%) suffered from vomiting and gastric reflex. Almost a third of the children suffered from abdominal pain (22.7%) and change in stool characteristics (34.1%). Only 11.4% of autistic children went to visit GI doctors (Further details in Table 2).

**Table 2:** The table shows the nutritional and diet characteristics of the children and the frequency of GIT symptoms (*N*=45)

The characteristic	Frequency	(%)
The BMI for the children:		
Underweight	17	37.8%
Healthy	19	42.2%
Overweight	4	8.9%
Obese	5	11.1%
Does the child have a diet regimen:		
Yes	19	42.2%
No	26	57.8%
Does having a diet program affect the child’s behavior?		
Yes	24	54.5%
No	20	45.5%
Does the child have food selectivity?		
Yes	34	57.56%
No	11	42.44%
Which food is your child selective for^a^:		
Noodle	39	17.4%
Rice	34	15.4%
Egg	29	13.2%
Potato	25	11.1%
Chicken and meat	20	8.8%
Lentil	18	8.2%
Tomato	10	4.4%
Apple	10	4.4%
Salad	5	2.2%
Does your child have an allergy to wheat?		
Yes	10	22.7%
No	35	77.8%
Does your child have an allergy to milk?		
Yes	10	22.2%
No	35	77.8%
Does the child take any autism supplements:		
No	26	57.8%
Yes	19	42.2%
Do you suffer from gastric reflex (burn)?		
Never	42	95.5%
One time in 2 months or more	0	0%
One to three times a month	0	0%
Once in a week	0	0%
Twice to four times a week	1	2.3%
Once a day	0	0%
Twice or more in a day	1	2.3%
Do you suffer from abdominal pain?		
Never	34	77.3%
One time in 2 months or more	4	9.8%
One to 3 times a month	0	0%
Once in a week	2	4.5%
Twice to four times a week	2	4.5%
Once a day	0	0%
Twice or more in a day	0	0%
Do you suffer from vomiting?		
Never	42	95.5%
One time in 2 months or more	0	0%
One to 3 times a month	1	2.3%
Once in a week	0	0%
Twice to four times a week	1	2.3%
Once a day	0	0%
Twice or more in a day	0	0%
Do you notice any change in the stool?		
Never	29	65.9%
One time in 2 months or more	4	9.1%
One to 3 times a month	4	9.1%
Once in a week	3	6.8%
Twice to four-time week	2	4.5%
Once a day	1	2.3%
Twice or more in a day	1	2.3%
Do you visit GIT doctors?		
Yes	5	11.4%
No	39	88.6%

^a^ The participants were allowed to select more than one choice.

### Factors affecting the behavioral changes scale and GIT symptoms scale

When doing univariate analysis for the sociodemographic factors against the behavioral changes and GIT symptoms frequency, there was only a significant difference between the different financial statuses of the guardians (*P*=0.018). It was found that high financial status is significantly different from the intermediate and low statuses based on behavioral changes (*P*=0.041 and 0.014, respectively) as it scaled the highest means of them (Further details in Table 1).

Diet regimen, allergies, and taking autism supplements were not found to be significant in univariate analysis of the behavioral changes scale and GIT symptoms frequency scale (Further details in Table 3).

**Table 3:** The table shows the univariate analysis of nutritional characteristics with the behavioral change scale and GIT symptoms frequency scale (*N*=45)

The characteristic	Behavioral change scale (Mean±SD)	*P* a	GIT symptoms scale (Mean±SD)	*P* b
Does the child take any autism supplements:		0.263		0.221
No	31.45±6.14		1.65±2.75	
Yes	27.80±7.33		2.71±4.03	
Does the child have a diet regimen :		0.674		0.136
Yes	30.27±6.19		2.47±3.15	
No	31.40±7.00		1.32±2.76	
Does your child have an allergy to wheat?		0.783		0.516
Yes	31.25±6.18		2.70±4.11	
No	30.47±6.68		1.56±2.55	
Does your child have an allergy to Milk?		0.547		0.134
Yes	31.78±2.96		3.60±4.43	
No	30.13±4.76		1.29±2.20	

a The scale for behavioral change in autistic children (Using Independent *T*-test).

b The scale for the frequency of GIT symptoms of Autistic children (using the Mann–Whitney test).

### Regression models for behavioral change scales and GIT symptoms scale

Multiple linear regression was performed for the GIT symptoms frequency scale. The R squire value was 0.475 while the adjusted R squire was 0.336. The regression model was significant (*P*=0.004). When plotting the sex, age, allergies, child autism supplements, BMI, and diet program status, no significant values were detected in the model (Further details in Table 4).

**Table 4:** The table shows multiple linear regression of factors affecting the GI symptoms frequency scale (*N*=45)

	Unstandardized coefficients		Standardized coefficients			95.0% CI for B	
The variable	B	Standard error	Beta	t	*P*	Lower bound	Upper bound
(Constant)	29.068	11.991		2.424	0.028	3.648	54.488
Sex	4.946	4.510	0.289	1.097	0.289	−4.615	14.506
Does the child take any autism supplements?	−0.876	3.376	−0.069	−0.259	0.799	−8.034	6.282
Does the child have a wheat allergy?	−0.474	4.950	−0.035	−0.096	0.925	−10.966	10.019
Does the child have a milk allergy?	3.060	5.469	0.234	0.559	0.584	−8.535	14.655
Age of child	0.332	0.660	0.147	0.503	0.622	−1.068	1.732
BMI	−0.136	0.411	−0.091	−0.331	0.745	−1.007	0.735
Residence	−2.092	2.943	−0.240	−0.0711	0.487	−8.329	4.146
Does the child follow a diet program?	−1.546	3.142	−0.0121	−0.492	0.629	−8.206	5.115

## Discussion

Autism spectrum disorder (ASD) is a complex neurodevelopmental condition that involves persistent problems and difficulty in social interactions, speech, and nonverbal communication, and restricted/repetitive behaviors compared to its matched child^1^. This study investigates the nutritional status of autistic children in Khartoum state, Sudan, by measuring their BMI and determining their percentile, as well as assessing whether autistic children follow a diet program and its impact on their behaviors. It also looked into the prevalence of gastrointestinal symptoms and whether they are related to following a diet plan and their relationship to specific food allergies (milk and wheat).

In our study, most of the participants were males(almost four-fifths of them), aged from 6 to 15 years old (school age) (73.3%) and living in intermediate financial status (71.1%) and this is the same as another study conducted in Khartoum, Sudan among autistic children^16^. More than half of the sample had both of their parents as their guardians (60%), and this is aligned with the fact that many parents derived fulfilment from providing care for their child with ASD^17^. When investigating the children’s history, almost more than 80% of them did not have any other medical condition or a family history of autism. Even autism was found to be associated with family history and with many other conditions, such as attention-deficit/hyperactivity disorder, according to a multinational study and a USA study, respectively^18,19^.

Almost more than half of the autistic children (57.8%) do not follow any specific diet program, even though more than half of them (54.5%) believe in the effect of diet programs on the behavior of the children. This percentage of adherence is higher than what happened in a study in the Slovak Republic where only a small percentage of the sample (21.2%) followed a specific diet program^20^. Only a fifth of the participants were overweight or obese (20.0%), and this can be due to high adherence to diet programs. This percentage is lower than the 39.0% indicated in the USA study for the same category and lower than the 64.1% indicated in the Brazilian study for overweight^21,22^. Regarding food selectivity and sensitives, more than half of the sample (57.6%) reported being selective about food with almost less than a third of the sample having an allergy to milk and wheat (22.2% for both milk and wheat). The percentage of food selectivity was lower than what is reported in another Slovakian study where 69.1% of the autistic children reported to have food selectivity^20^. Moreover, another clinical trial authors stated autistic children tend to be ‘Picky eaters’ with a preference for only certain foods^23^. In addition, more than half of the autistic children (57.8%) are not given any autistic supplements which is lower than the food supplement usage percentage in another Slovakian study (66.7%)^20^. These discrepancies between our findings and the Slovakian study could be attributed to the measures and scales adopted, as well as methodological variations in terms of sample size and sample selection.

In our study, there was a small percentage of children suffering GIT symptoms ranging from 4.5% for vomiting and gastric reflex to 22.7% for abdominal pain. This opposes the high prevalence of GIT symptoms among autistic samples in the Slovak Republic and the US which were 88.9 and 61.0%, respectively^20,24^. The two-way connection between mental health and gastrointestinal symptoms highlights the need for holistic treatment. Overall, a thorough, patient-centered strategy that combines research and collaborative efforts is essential for enhancing outcomes and alleviating the societal impact of GI disorders^25^.

Regarding the factors affecting the behavioral changes scale, only the functional status was found to be significant (*P*-value 0.018). It was found that only high financial status is significantly different from the intermediate and low financial statuses (*P*=0.041 and 0.014, respectively). This result is justifiable as according to another study in China, having an autistic child increases the odds of disturbing with parents’ work (OR:15.936, *P*>0.001) and decreases the odds of living in a high financial status (OR=−1.271, *P*>0.001)^26^. This finding gives an insight into the significance of dealing with autism with a holistic approach encompassing the medical and psychological aspects, as well as the socioeconomical aspect. A previous case–control study was carried out in Bangladesh, to evaluate the relationship between socioeconomic status and the risk of developing autism spectrum disorder (ASD). Findings indicated that a higher socioeconomic status, a father’s advanced education level (master’s degree or higher), fathers aged 22–35 years, and living in a nuclear family were significantly associated with reduced odds of ASD compared to healthy controls^27^. Such findings have important implications for understanding the factors that contribute to behavioral changes. It suggests that individuals’ functional abilities and financial situations can impact how they behave or respond to certain situations. By identifying these significant factors, interventions or strategies can be developed to target and address these specific areas to promote positive behavioral changes. Neither the age, sex, diet regiment, allergies to milk and wheat, taking autism supplements, or family history was found to be significant with behavioral changes in univariate analysis. In contrast, food allergies were found to be significantly associated with autism in a US study after adjusting for sociodemographic factors (*P*>0.001)^28^.

Talking about factors that affect GIT symptoms frequency, the multilinear regression did not yield any significant findings when inserting sex, age, allergy to wheat, milk allergy, BMI, diet program status, and taking autism supplements. These findings are opposed to two other studies. One of these studies is in China, which found that not having a diet program was an independent factor associated with GI symptoms (adjusted standardized β: 0.231, 95% CI: 0.063–0.400, *P*=0.008)^29^. The other study was in Iraq which found a significant correlation between eating habits and GIT symptoms (*P*=0.001)^30^. This discrepancy highlights the importance of taking diet and eating habits into consideration when assessing factors affecting GIT in children with autism.

This study can be viewed in light of some limitations which include the cross-sectional study and the small sample size. In addition, the majority were not enrolled in a specific diet program, hence sabotaging the assessment of any effect reported from diet programs. Therefore, we recommend that interventional studies with specific diet programs must be implemented to assess the direct impact of the diet programs on behavior and gastrointestinal symptoms. A previous study employed a precision nutrition strategy, incorporating genetic profiles, microbiome composition, and physiological parameters to develop personalized dietary plans. This digitalized approach provides cost benefits through increased efficiency, scalability, and data analysis, along with the advantages of personalization, real-time monitoring, continuous support, and behavior modification, making it superior to traditional methods^31^. Additionally, further research involving multidisciplinary experts’ teams such as pediatricians, dietitians, psychologists, gastroenterologists, and nutritionists should be conducted because the collaboration across various fields will contribute to a holistic understanding of the complex interactions between diet, behavior, and gastrointestinal health in children with autism. Finally, we encouraged every center to employ a nutritionist to educate mothers on the importance of consuming recommended foods, particularly during early infancy, to promote better outcomes in adulthood.

## Conclusion

The majority of participants exhibited good nutritional health as indicated by their BMI, with a noteworthy minority experiencing underweight conditions. The greater portion of the participants did not follow a specific diet program, most of them revealed a high degree of food selectivity. In addition, a minority of participants had an allergy to milk and wheat. Moreover, some of the autistic children reported suffering from vomiting, gastric reflux, abdominal pain as well as changes in stool characteristics. The study revealed no significant association between the study variables in multivariant analysis.

## Ethical approval

Ethical consent was obtained from the ethics committee of the Faculty of Medicine, University of Khartoum. Permission was then taken from the autism centers’ administration.

## Consent

Written informed consent was obtained from the patient’s parents/legal guardian for publication and any accompanying images. A copy of the written consent is available for review by the Editor-in-Chief of this journal on request.

## Source of funding

The study did not receive funding support.

## Author contribution

S.O.M.E., S.O.M.E., M.H.M.E., W.Y.A.S., and L.M.K.: wrote and drafted the first version of the manuscript and conceptualized it; D.R.H.A., L.H., K.A.H.M.A., and G.E.M.A.: visualized, validated, conceptualized, and wrote and drafted the manuscript’s final version and critically reviewed and edited the initial draft and proofread, and edited the manuscript’s final version. All authors reviewed and approved the final manuscript.

## Conflicts of interest disclosure

The authors declare no conflict of interest.

## Research registration unique identifying number (UIN)

1. Name of the registry: it is an observational study and the research registration was not required.
2. Unique identifying number or registration ID: It is an observational study and the research registration was not required.
3. Hyperlink to your specific registration (must be publicly accessible and will be checked): it is an observational study and the research registration was not required.

## Guarantor

Khabab Abbasher Hussien Mohamed Ahmed, Faculty of Medicine, University of Khartoum, 11111 Khartoum, Sudan. E-mail: Khabab9722@gmail.com, ORCID: https://orcid.org/0000-0003-4608-5321.

## Data availability statement

The data that support the findings of this study is available from the corresponding author upon reasonable request.

## Provenance and peer review

Externally peer-reviewed, not commissioned.
